# Potential contributions of root decomposition to the nitrogen cycle in arctic forest and tundra

**DOI:** 10.1002/ece3.3522

**Published:** 2017-11-15

**Authors:** Sabrina Träger, Ann Milbau, Scott D. Wilson

**Affiliations:** ^1^ Department of Botany Institute of Ecology and Earth Sciences University of Tartu Tartu Estonia; ^2^ Research Institute for Nature and Forest INBO Brussels Belgium; ^3^ Department of Ecology and Environmental Science Climate Impacts Research Centre Umeå University Abisko Sweden; ^4^ Department of Biology University of Regina Regina SK Canada

**Keywords:** home‐field advantage, litter quality, minirhizotron, nitrogen content, plant litter, reciprocal transplant experiment, root production

## Abstract

Plant contributions to the nitrogen (N) cycle from decomposition are likely to be altered by vegetation shifts associated with climate change. Roots account for the majority of soil organic matter input from vegetation, but little is known about differences between vegetation types in their root contributions to nutrient cycling. Here, we examine the potential contribution of fine roots to the N cycle in forest and tundra to gain insight into belowground consequences of the widely observed increase in woody vegetation that accompanies climate change in the Arctic. We combined measurements of root production from minirhizotron images with tissue analysis of roots from differing root diameter and color classes to obtain potential N input following decomposition. In addition, we tested for changes in N concentration of roots during early stages of decomposition, and investigated whether vegetation type (forest or tundra) affected changes in tissue N concentration during decomposition. For completeness, we also present respective measurements of leaves. The potential N input from roots was twofold greater in forest than in tundra, mainly due to greater root production in forest. Potential N input varied with root diameter and color, but this variation tended to be similar in forest and tundra. As for roots, the potential N input from leaves was significantly greater in forest than in tundra. Vegetation type had no effect on changes in root or leaf N concentration after 1 year of decomposition. Our results suggest that shifts in vegetation that accompany climate change in the Arctic will likely increase plant‐associated potential N input both belowground and aboveground. In contrast, shifts in vegetation might not alter changes in tissue N concentration during early stages of decomposition. Overall, differences between forest and tundra in potential contribution of decomposing roots to the N cycle reinforce differences between habitats that occur for leaves.

## 1 INTRODUCTION

Fine root production and mortality are major components of vegetation effects on ecosystem functioning such as nitrogen (N) cycling (Pendall, Rustad, & Schimel, 2008; Schmidt et al., 2011). Factors altering N dynamics (e.g., mineralization and immobilization) during decomposition of plant tissue include the quantity of tissue (i.e., production; Jo, Fridley, & Frank, 2016), the quality of tissue (i.e., tissue N concentration; Cornwell et al., 2008), as well as the ability of decomposer communities to degrade plant tissue (Keiser, Keiser, Strickland, & Bradford, 2014). These factors are well‐quantified for aboveground plant tissue but not for belowground tissue. Yet, in many terrestrial ecosystems, the majority of plant biomass is belowground, with arctic ecosystems exhibiting up to 80% of plant biomass belowground (Iversen et al., 2015; Mokany, Raison, & Prokushkin, 2006). Arctic ecosystems are among the most sensitive to environmental changes caused by climate warming (ACIA, 2005; Hobbie et al., 2017). These changes include shifts in dominant vegetation, which are likely to alter N cycling by changing the quantity and quality of plant tissue. The effects of vegetation change on belowground plant contributions to N cycling are almost entirely unknown.

One of the most consistent changes in arctic vegetation in recent decades has been the increased cover of woody functional groups (i.e., shrubs and trees; Hallinger & Wilmking, 2011; Myers‐Smith et al., 2015; Rundqvist et al., 2011). This increase in woody vegetation is associated with both indirect and direct effects on soil N cycling. Indirect effects of woody expansion include an increase in soil temperature (Hallinger, Manthey, & Wilmking, 2010), and possible changes in soil moisture. Direct effects of woody expansion on N cycling are likely related to traits such as tissue mass production and [N] (Buckeridge, Zufelt, Chu, & Grogan, 2010; Myers‐Smith et al., 2011). Greater aboveground production (Mendoza‐Ponce & Galicia, 2010) and higher litter quality (i.e., higher [N]) in forest than in herbaceous vegetation (Steinaker & Wilson, 2005; Sturm, Douglas, Racine, & Liston, 2005) may contribute to greater potential N input (Clark et al., 2001; Norris, Blair, Johnson, & McKane, 2001) and thus increased rates of soil N cycling in forest (Jackson, Banner, Jobbagy, Pockman, & Wall, 2002). Fine roots (root diameter <2.0 mm) might also influence N cycling due to their generally high production and high [N] (Pregitzer et al., 2002; Steinaker & Wilson, 2005), but their relative contribution to the N cycle in herbaceous and woody arctic vegetation is unknown.

Differences between herbaceous and woody vegetation in root production may have consequences for N cycling, comparable to those for aboveground tissue, as tissue quantity is an important factor driving potential N input (Jo et al., 2016). Studies of root mass production in the Arctic often consider only one habitat (forest: Finér, Ohashi, Noguchi, & Hirano, 2011; Hansson, Helmisaari, Sah, & Lange, 2013; tundra: Sullivan et al., 2007), and studies directly comparing those two habitats are underrepresented. A meta‐analysis by Freschet et al. (2013) suggests that root mass input is greater in forest than tundra, but conclusions for the potential N input of roots comparing forest and tundra are lacking. We hypothesize that the greater aboveground tissue production in arctic forest might be accompanied by greater root production, resulting in greater potential N input of roots during decomposition in forest.

Tissue [N] is likely to be important for influencing the potential N contribution of roots (Balogianni, Wilson, Farrell, & MacDougall, 2015; Castro‐Díez, Godoy, Alonso, Gallardo, & Saldana, 2014), and root [N] varies with traits such as diameter and color: [N] decreases with increasing root diameter (Bahn, Knapp, Garajova, Pfahringer, & Cernusca, 2006; Pregitzer, Kubiske, Yu, & Hendrick, 1997; Steinaker & Wilson, 2005). Young, small, white roots are often more physiologically active and have a higher nutrient uptake capacity than brown roots (Gu, Wei, Wang, Dong, & Wang, 2015). Physiological activity suggests that white roots might have greater [N] than brown roots (Baldi, Wells, & Marangoni, 2010). However, the accumulation of N‐rich secondary compounds (i.e., tannin or lignin) in the roots’ epidermis over time (MacDougall & Wilson, 2011) might lead to greater [N] in older brown than younger white roots. We expect differences in the root potential N input in contrasting habitats (forest and tundra) to vary with root diameter and color.

One of the most important factors driving decomposition of plant tissue is the decomposer microbial community (Ayres et al., 2009). Several studies suggest that litter decomposes faster in its habitat of origin than in other habitats (de Toledo Castanho & de Oliveira, 2008; Vivanco & Austin, 2008) because decomposers of any community work most efficiently with litter from their own community (a “home‐field advantage,” Gholz, Wedin, Smitherman, Harmon, & Parton, 2000). Consequently, the interaction between tissue quality and the associated decomposer community of contrasting habitats (forest and tundra) may be of great importance (Cornwell et al., 2008). Decomposition of woody vegetation expanding in herbaceous vegetation might be limited by the loss of the home‐field advantage. Therefore, we tested whether the habitat of origin (forest or tundra) and the habitat where decomposition occurred (forest or tundra) affected changes in [N] in roots and leaves.

Here, we examined the potential N input of roots and leaves in arctic forest and tundra, by measuring tissue production, [N], and changes in [N] caused by decomposition. We defined potential N input as the amount of N in plant tissue likely to be contributed to the N cycle during decomposition. We also tested for a home‐field advantage using a reciprocal litter transplant experiment. We examined current forest and tundra, to gain insights into possible changes in the N cycle in tundra caused by increased cover of woody vegetation related to climate change. We tested whether (1) the potential N input of roots and leaves is greater in forest than tundra; (2) the potential N input varies with root traits like diameter and color; and (3) changes in N concentration of roots and leaves in forest and tundra during 1 year of decomposition varies with habitat of origin (forest or tundra) and the habitat in which decomposition occurs (forest or tundra).

## 2 MATERIALS AND METHODS

### 2.1 Potential N input in forest and tundra

#### 2.1.1 Potential N input: Study sites

We studied potential N input of roots and leaves in deciduous birch forest and herbaceous‐perennial tundra about 200 km inside the Arctic Circle near Abisko, Sweden. The mean annual temperature measured at Abisko is 0.1°C (1961–2013; SMHI, 2014). About one‐third of the mean annual precipitation occurs in summer. Snow cover extends from early October to mid‐June. The soil in the study area is derived from a schist–limestone–quartzite mixture (SGU, 2015). The forest habitat was near the mouth of Nissonjohka river, 400 m above sea level (a.s.l.) (68°20′N 18°46′E). The tundra habitat was in Kärkevagge valley, 720 m a.s.l. (68°24′N 18°19′E). These two sites were chosen because they have locally rare, relatively deep soils that allow installation of minirhizotron tubes 1 m long belowground at 45°. The mean annual precipitation near the forest is 310 mm (1913–2000; Kohler, Brandt, Johansson, & Callaghan, 2006), and near the tundra 1,000 mm (1961–2013; SMHI, 2014). Dominant species in the forest habitat are *Betula pubescens subsp. czerepanovii* (N.I. Orlova) Hämet‐Ahti, *Deschampsia flexuosa* (L.) Trin., *Vaccinium* spp., and *Empetrum nigrum* L., and, in the tundra habitat, *Carex* spp., *Bistorta vivipara* (L.) Delarbre, *Pyrola minor* L., *Ranunculus acris* L., and *Viola biflora* L.

#### 2.1.2 Potential N input: Belowground production, N concentration, and N input

We calculated the potential N input of fine roots by measuring root length production, multiplying length production by specific root weight to get root mass production (hereafter root production), and multiplying root production by root [N].

We examined fine root production at five randomly chosen locations in forest and tundra habitats. Locations at each habitat were spread over an area *c*. 500 m in diameter with a minimum separation of 30 m. At each location, one transparent plastic tube (diameter = 5 cm, length = 120 cm, clear cellulose acetate butyrate) was installed in 2010 at a 45° angle to the soil surface with one end emerging from the soil surface to allow access for a minirhizotron camera. The protruding part of the tube was capped and covered with black tape to exclude sunlight. It has been argued that rhizotron tubes pose an unnatural barrier to root growth, producing results that differ from root growth in bulk soil (Rytter & Rytter, 2011). However, soils in our study area are very stony (Rubensdotter, 2002), so that the behavior of roots that encounter physical barriers is relevant in our study system and in lithosols in general.

We used a minirhizotron camera (Bartz Technology, Santa Barbara, CA, USA) to repeatedly collect 30 images (each 18 × 13.5 mm), each 13.5 mm apart, of the upper surface of each tube, from the soil surface to *c. *65 cm depth. This soil depth includes the majority of fine roots in arctic soils (Iversen et al., 2015; Jackson et al., 1996). We recorded images biweekly from mid‐June to beginning of September of 2013 and 2014. This time interval captures root production without missing root mortality in our study area (Balogianni, Blume‐Werry, & Wilson, 2016). We measured the total length of live roots (excluding root hairs) in minirhizotron images using Rootfly (version 2.0.2.; Clemson University, 2011) and summed root length per tube. We considered a root to be alive if it was white or brown (Hendrick & Pregitzer, 1992). We recorded fine root length in five diameter classes (≤0.1, 0.1–0.2, 0.2–0.5, 0.5–1, 1–2 mm) and two color classes (brown and white) because [N] potentially varies with these traits (Steinaker & Wilson, 2005). Root length production was calculated as the increase in root length (elongation of existing roots and the appearance of new roots) between the first and last sample of each growing season for each tube. Using the first and last samples gave the same result as summing over each biweekly sample period due to the low mortality of roots in this system (Balogianni et al., 2016; S. Träger, unpublished data).

We determined root mass production (g root m^−2^ of image) by multiplying root length production by specific root weight (SRW = root mass per cm of root length, i.e., g root cm^−1^ root) for each diameter and color class. SRW was determined based on root samples in forest and tundra from ten soil cores, with two pooled soil cores (diameter = 2.5 cm, depth = 60 cm) per location, collected in June 2014, resulting in five soil cores per habitat. Roots were separated into diameter and color classes, washed, measured for length, dried for 24 hr at 80°C, and weighed. Root mass was estimated by using linear models of SRW for each diameter and color class in each habitat.

We determined [N] of fine roots collected in beginning of September in 2013. We took three soil cores (diameter = 5 cm, depth = 60 cm), at each location, pooled them, and sorted roots by diameter and color, as above. Sorted roots were washed and dried for 48 hr at 60°C. Dried root samples were ground in a Mixer Mill MM301 (Retsch GmbH, Germany). We measured total N and carbon (C) concentration in 5 mg of ground roots using an Isotope Ratio Mass Spectrometer (EA‐IRMS; ThermoFischer; Swedish University of Agricultural Sciences, Umeå, Sweden).

Potential N input from annual root production was calculated by multiplying mass production of each root diameter and color class by its tissue [N].

For more information on the aboveground vegetation sampling procedure please see Supporting Information.

#### 2.1.3 Potential N input: Statistical analysis

Variation in potential N input, root and leaf production, and [N] and C:N ratio of leaves was examined using two‐way mixed‐effect model ANOVAs with year of sampling and habitat as fixed factors, and location depending on sampling year as random factor (function lme, R package “nlme”; R Development Core Team, 2015). Year as factor was included to test if production varies among years. Variation in [N] and C:N ratio of roots was tested using one‐way ANOVA with habitat as fixed factor. Post hoc tests for two‐way mixed‐effect model ANOVAs were performed using least square means with the function lsmeans from the R package “lsmeans” (Lenth, 2016), with the adjustment method “Tukey.”

We tested variation in potential N input and production among root diameter and color classes using four‐way mixed‐effect model ANOVAs with habitat, diameter, color, and year as fixed factors, and location depending on sampling year as random factor. Variation in [N] and C:N ratio among root diameter and color classes was tested using three‐way ANOVAs with habitat, diameter, and color as fixed factors (function aov, R package “aov”; R Development Core Team, 2015). For clarity, we present results of this analysis separated in diameter and color classes, but also in an overall ANOVA table. Independency between color and diameter was ascertained by means of a contingency table. Data were transformed to obtain normality if necessary.

### 2.2 Changes in tissue N concentration during decomposition in forest and tundra

#### 2.2.1 Decomposition: Study sites

We examined whether N contribution of roots and leaves to the soil during early stages of decomposition varies with the habitat where decomposition occurs (forest or tundra) and the habitat origin of the tissue (forest or tundra). We studied decomposition of roots and leaves in three areas near Abisko: Låktajåkka (68°25′N 18°21′E), Nuolja (68°21′N 18°45′E), and Suorovari (68°19′N 19°10′E). In each area, we studied two habitats (deciduous birch forest and herbaceous‐perennial tundra) with four locations at each habitat. The forest habitats were at 400 m a.s.l., and the tundra habitats were at 650 m a.s.l. Dominant species in forest habitats were *Betula pubescens subsp. czerepanovii* (N.I. Orlova) Hämet‐Ahti., *Deschampsia flexuosa* (L.) Trin., *Geranium sylvaticum, Milium effusum,* and *Rubus saxatilis*, and, in tundra habitats, *Anthoxantum odoratum* L., *Bistorta vivipara* (L.) Delarbre, and *Viola biflora* L. (J. Lembrechts, unpublished data).

#### 2.2.2 Decomposition experiment

We measured [N] of fine roots and leaves in forest and tundra before and after 1 year of decomposition. A reciprocal decomposition experiment allowed tissue to decompose both in its own habitat (“home”) as well as in the other habitat (“away”).

We collected fine roots in three soil cores (diameter = 2.5 cm, depth = 20 cm, separated by *c*. 200 cm) per location and pooled these by location, end of August 2014. We sorted out fine roots of two diameter classes (≤0.2 and >0.2 mm) because they potentially vary in [N] (Steinaker & Wilson, 2005). One subsample from each diameter class per plot was oven‐dried (60°C, 48 hr) for initial nutrient analysis ([N] and [C]). Two root subsamples from each root diameter class per location were air‐dried for 48 hr, weighed (*c. *100 mg), and placed in polyester litterbags (7 × 9 cm, mesh size of 0.3 mm). The mesh size was chosen for both roots and leaves to allow comparable access for microbes and micro‐invertebrates to the substrates and due to the lack of large soil invertebrates in this arctic area (Cornelissen et al., 2007; Freschet, Aerts, & Cornelissen, 2012). One subsample decomposed in its own location (home), and the other decomposed in its respective location in the other habitat (away). For information on leaf sampling please see Supporting Information.

We field‐incubated aboveground and belowground tissues in a reciprocal design for 1 year, beginning end of August 2014. Each location per habitat per area received two root samples (roots ≤0.2 mm, roots >0.2 mm) and one leaf sample from the original habitat (home) and two root samples and one leaf sample from the respective other habitat (away). Litterbags were buried 10 cm deep. After 1 year of decomposition, soil, soil fauna, and other material were removed from the decomposed litter by sorting and rinsing with tap water. Litter samples were oven‐dried (60°C, 48 hr) and weighed, and total [N] and [C] was determined as described above. Due to difficulties with mass measurements after decomposition, it was not possible to calculate mass loss for our decomposition samples. However, the omission of mass loss data does not invalidate our results and conclusions (see Discussion and Supporting Information “Total N release dynamics”).

We calculated changes in [N] after 1 year of decomposition for each tissue type at each location per habitat, depending on the origin of the tissue type, by subtracting [N] after decomposition from initial [N] before decomposition.

#### 2.2.3 Decomposition: Statistical analysis

Variation in [N] and C:N ratio before and after decomposition and change in [N] after 1 year of decomposition were examined using two‐way and three‐way mixed‐effect model ANOVAs, respectively, with tissue type (roots ≤0.2 mm, roots >0.2 mm, leaves), habitat origin (home or away), and incubation habitat (forest or tundra) as fixed factors and location as random factor (function lme, R package “nlme”; R Development Core Team, 2015). The test method for mixed‐effect ANOVAs was maximum likelihood. Post hoc tests were performed using least square means with the function lsmeans from the R package “lsmeans” (Lenth, 2016), with the adjustment method “Tukey.” Data were transformed to obtain normality if necessary. All of the statistical analyses were implemented in R 3.2.3 (R Development Core Team, 2015).

## 3 RESULTS

### 3.1 Potential N input in forest and tundra

**Figure 1:**
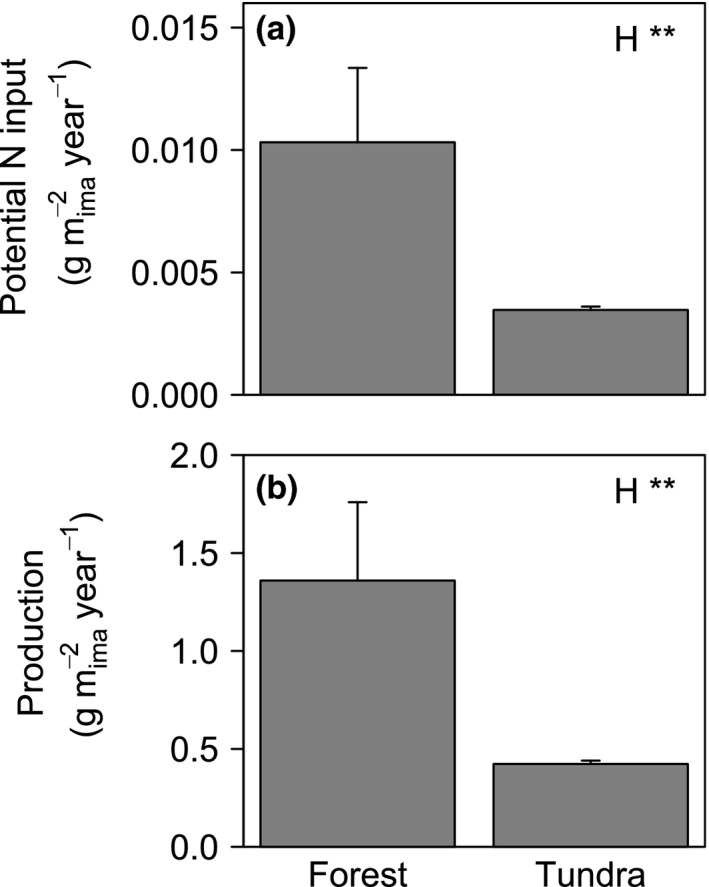
Mean (±*SD*) potential nitrogen (N) input (a) and mass production (b) of roots in forest and tundra. Root potential N input and production are in g m^−2^ minirhizotron image year^−1^. Effect: habitat (H): forest or tundra. **p < .01

Potential N input (mass production multiplied by tissue [N]) of roots in forest exceeded that in tundra by a factor of two (*F*_1,10_ = 11.90; *p* < .01; Figure 1a). This was associated with much greater root production in forest than tundra (*F*_1,12_ = 18.15; *p* < .01; Figure 1b). Year had no significant (*p* > .05) effect on potential N input or production of roots (N input: *F*_1,10_ = 0.94; *p* = .36; production: *F*_1,12_ = 0.05; *p* = .84). In contrast, root [N] did not vary significantly between habitats (forest: 0.76% ± 0.19%, mean ± *SD*; tundra: 0.82% ± 0.17%; *F*_1,90_ = 1.67; *p* = .20). The C:N ratio of roots was significantly greater in forest than tundra (forest: 55.13 ± 18.86; tundra: 41.91 ± 15.26; *F*_1,88_ = 13.43; *p* < .001).

**Figure 2:**
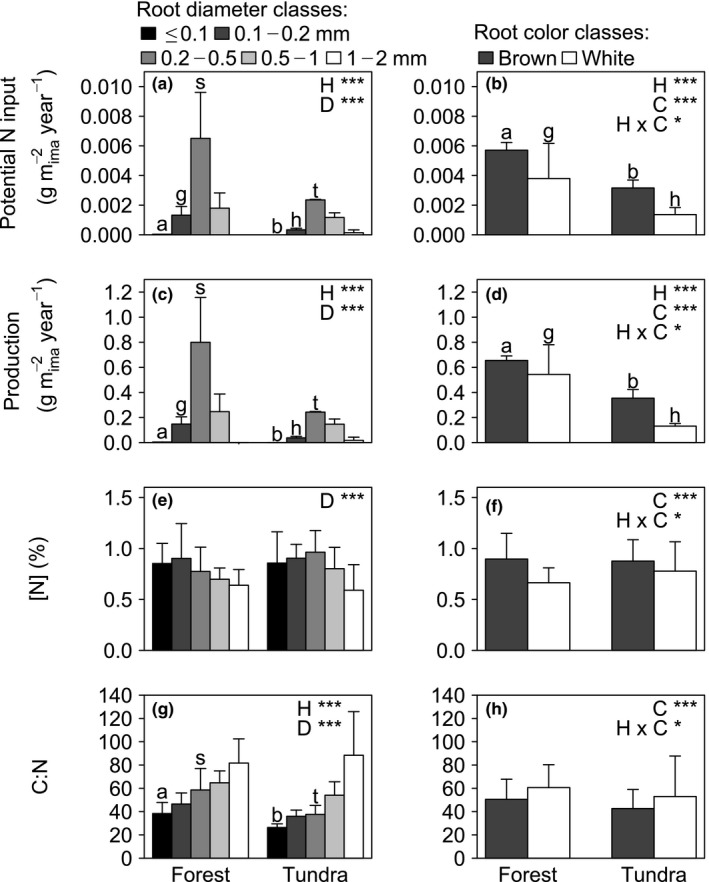
Mean (±*SD*) potential N input, mass production, [N], and carbon (C):N ratio of roots for different root diameter classes (a, c, e, g) and color classes (b, d, f, h) in forest and tundra. Root potential N input and production represent values in g m^−2^ minirhizotron image year^−1^. Values per diameter classes are averaged over color classes. Values per color classes are averaged over diameter classes. Effect: habitat (H): forest or tundra; diameter (D): root diameter ≤0.1, 0.1–0.2, 0.2–0.5, 0.5–1, 1–2 mm; Color (C): root color brown or white. ***p < .001; *p < .05. Means within the same diameter or color class marked with a–b, g–h, or s–t are significantly different in forest and tundra (Tukey HSD,* p* < .05). Missing indication of significance indicates no significant effect. For clarity, we present results separated per diameter and color classes. Complete statistical results of four‐way ANOVAs for potential N input, production, [N], and C:N ratio of roots are presented in Table 1

Potential N input also varied significantly among root diameter and color classes (Figure 2a,b; Table 1). The highest potential N input in both forest and tundra occurred for roots with a diameter of 0.2–0.5 mm, whereas roots ≤0.1 mm and roots >1 mm potentially contributed very little (Figure 2a). Root potential N input was higher in forest than tundra only for roots with a diameter ≤0.5 mm (Figure 2a). There were no significant interactions involving root diameter and habitat (Table 1), suggesting that forest and tundra have similar distributions of potential N input from roots of different diameters. In contrast, a significant interaction occurred between root color and habitat because brown roots had greater potential N input than white roots, but significantly so only in tundra (Figure 2b).

**Table 1:** *F*‐values from ANOVAs of root potential nitrogen (N) input, mass production, [N], and carbon (C):N ratio. Effects: habitat: forest or tundra; diameter: root diameter ≤0.1; 0.1–0.2; 0.2–0.5; 0.5–1; 1–2 mm; Color: root color brown or white roots; Year: 2013 or 2014. *df* and residual *df* are shown for all variables tested (root potential N input, root production, root [N], and root C:N ratio). Values in parentheses refer to *df* or residual *df* of root [N] and root C:N ratio

Effect	*df*	Residual *df*	Root potential N input (g m^−2^ image year^−1^)	Root production (g m^−2^ image year^−1^)	Root [N] (%)	Root C:N
Habitat	1	57 (73)	19.12^c^	23.41^c^	2.03	40.28^c^
Diameter	4	57 (73)	43.75^c^	44.64^c^	9.50^c^	56.83^c^
Color	1	57 (73)	29.70^c^	23.48^c^	20.38^c^	13.60^c^
Year	1	57 (—)	0.16	0.31	—	—
Habitat × Diameter	3 (4)	57 (73)	2.84	2.56	0.91	1.29
Diameter × Color	2 (4)	57 (73)	6.75**	8.15^c^	3.27*	4.50**
Color × Habitat	1	57 (73)	5.31*	7.91*	3.97*	4.00*
Diameter × Color × Habitat	2 (4)	57 (73)	0.63	0.72	0.96	1.03

a *p* < .05.

b *p* < .01.

c *p* < .001.

Root production also varied significantly among root diameter and color classes (Figure 2c,d; Table 1). The greatest production in both forest and tundra occurred in roots with a diameter of 0.2–0.5 mm. Root production was higher in forest than tundra only for roots with a diameter ≤0.5 mm (Figure 2c). Similar to potential N input, there was no significant interaction between root diameter and habitat (Table 1). A significant interaction occurred between root color and habitat because brown roots had significantly greater root production than white roots, but only in tundra (Figure 2d). Year had no significant effect on potential N input or production of roots separated by diameter (Table 1).

Root [N] varied significantly among root diameter and color classes (Figure 2e,f; Table 1). Root [N] in both forest and tundra was generally lowest in roots with the greatest diameter (Figure 2e). A significant interaction between color and habitat occurred because brown roots had significantly higher [N] than white roots, but only in forest (Figure 2f).

Root C:N ratio varied significantly with diameter and color (Figure 2g,h; Table 1). Root C:N ratio was greatest in roots with greater diameter in both forest and tundra (Figure 2g). Root C:N ratio was higher in forest than tundra only for roots with a diameter ≤0.1 and 0.2–0.5 mm (Figure 2g). Root C:N ratio showed a significant interaction between color and habitat because white roots had an higher C:N ratio than brown roots only in tundra (Figure 2h).

Potential N input from leaves was significantly greater in forest than tundra (Table 2). This resulted from a significantly greater production in forest than tundra, even though [N] of leaves was significantly greater in tundra than forest (Table 2). Also, the C:N ratio of leaves was significantly greater in forest than tundra (Table 2). Year had no significant effect on potential N input, production, [N], or C:N ratio of leaves (Table 2).

**Table 2:** Mean (±*SD*) potential N input, mass production, [N], and C:N ratio for different aboveground tissues in forest (understorey and leaves) and tundra. *F*‐values_(habitat)_ and *F*‐values_(year)_ indicate significant differences between habitats (forest and tundra), and year of sampling (2013 and 2014) of leaf potential N input, leaf production, leaf [N], and leaf C:N

Habitat	Component	Leaf potential N input (g m^−2^ year^−1^)	Leaf production (g m^−2^ year^−1^)	Leaf [N] (%)	Leaf C:N
Forest	Understorey	0.51 (0.45)	45.53 (30.91)	1.03 (0.19)	44.73 (7.27)
Forest	Leaves	5.81 (2.94)	440.90 (192.68)	1.24 (0.19)	41.49 (6.68)
Tundra	Herbs	0.88 (0.39)	60.71 (25.75)	1.44 (0.17)	31.97 (3.83)
Total forest		6.32 (3.36)	486.43 (243.26)	1.13 (0.22)	43.19 (7.00)
Total tundra		0.88 (0.39)	60.71 (25.75)	1.44 (0.17)	31.97 (3.83)
*F*‐value_(habitat)_		35.43^b^	54.40^b^	14.41**	21.55^b^
*F*‐value_(year)_		0.48	0.14	0.95	2.61

a *p* < .01.

b *p* < .001.

### 3.2 Changes in tissue N concentration during decomposition in forest and tundra

**Figure 3:**
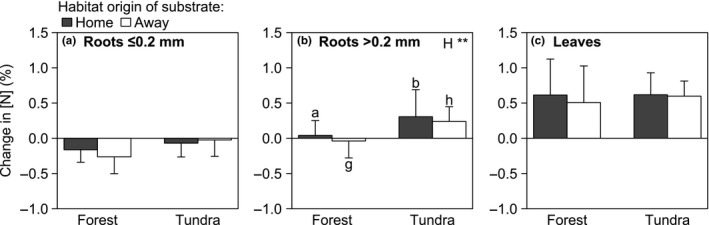
Mean (±*SD*) change in nitrogen (N) concentration (%) of roots with diameter ≤0.2 mm (a), roots with diameter >0.2 mm (b), and leaves (c) after 1 year of decomposition in forest and tundra, for tissue from the same habitat (“home,” shaded bars) and from the other habitat (“away,” open bars). Change in [N] was calculated as the difference of initial [N] before decomposition and [N] after 1 year of decomposition. Positive values of change in [N] indicate N accumulation, negative values indicate N loss. Effect: Habitat (H): forest or tundra. **p < 0.01. Means within the same origin of substrate with a–b, or g–h are significantly different in forest and tundra (Tukey HSD test, *p* < .05). Missing indication of significance indicates no significant effect. For clarity, we present results separated by tissue type. Results of three‐way ANOVAs for change in [N] of roots after 1‐year decomposition are presented in Table 3

Changes in [N] after 1 year of decomposition did not vary significantly with habitat origin (forest or tundra; Figure 3; Table 3), but did vary significantly among tissue types: roots ≤0.2 mm in diameter tended to decrease in [N], roots >0.2 mm in diameter both decreased and increased in [N], and leaves increased in [N] (Figure 3). A significant interaction between incubation habitat and tissue type occurred because only roots >0.2 mm showed a significant difference in change in [N] between habitats (Figure 3b, Table 3). Significantly more N was accumulated in roots >0.2 mm in tundra than forest. Roots ≤0.2 mm in diameter showed a similar but nonsignificant response (Figure 3a). Leaves accumulated N in both forest and tundra (Figure 3c). The significant interaction between tissue type and incubation habitat also suggests that differences between tissue types depended on the incubation habitat: [N] of roots ≤0.2 mm decreased in both forest and tundra (Figure 3a), whereas roots >0.2 mm accumulated N most strongly in tundra (Figure 3b), and leaves accumulated N in both incubation habitats (Figure 3c).

**Table 3:** *F*‐values from ANOVAs of change in [N], [N], and C:N ratio after one year of decomposition depending on the habitat, substrate and origin of substrate. Effects: Habitat: forest or tundra; Substrate: roots ≤0.2 mm, roots >0.2 mm or leaves; Origin: substrate from home or away habitat. *df* and residual *df* are shown for all variables tested (change in [N], [N], and C:N ratio). Values in parentheses represent residual *df* of change in [N]

Effect	*df*	Residual *df*	Change in [N] (%)	[N] (%)	C:N
Habitat	1	129 (126)	9.35**	1.61	1.56
Substrate	2	129 (126)	96.62***	312.7***	195.53***
Origin	1	129 (126)	1.03	1.09	0.72
Habitat × Sub	2	129 (126)	5.25**	0.13	0.32
Habitat × Ori	1	129 (126)	0.49	0.61	1.05
Sub × Ori	2	129 (126)	0.09	0.07	0.1
Habitat × Sub × Ori	2	129 (126)	0.22	0.14	0.09

***p < .001; **p < .01

N concentration before decomposition did not vary significantly between habitats (*F*_1,65_ = 1.71; *p* = .20), but differed significantly among tissue types (*F*_2,65_ = 64.84; *p* < .001; leaves: 2.11% ± 0.21%; roots ≤0.2 mm: 1.50% ± 0.28%; roots >0.2 mm: 1.10% ± 0.21%). Similarly, [N] after decomposition differed significantly among tissue types (Table 3). N concentration after decomposition was significantly greater in leaves (2.70% ± 0.05%), followed by roots ≤0.2 mm (1.37% ± 0.06%), and roots >0.2 mm (1.24% ± 0.07%).

The C:N ratio before decomposition varied significantly among tissue types (*F*_2,65_ = 45.23; *p* < .001). The C:N ratio before decomposition was greater in roots >0.2 mm (44.08 ± 10.33), followed by roots ≤0.2 mm (27.95 ± 5.49), and leaves (22.14 ± 2.76). Similarly, the C:N ratio after decomposition differed significantly among tissue types (Table 3) and was greater in roots >0.2 mm (41.94 ± 2.81), than in roots ≤0.2 mm (32.46 ± 3.67), and leaves (19.25 ± 2.30).

## 4 DISCUSSION

The potential input of roots to the N cycle was twofold greater in forest than tundra, resulting from greater root mass production in forest and no difference in [N] of roots between habitats. The potential N input from leaves was also greater in forest than tundra, due to a greater mass production in forest, even though [N] of leaves was greater in tundra. During early stages of decomposition (1 year) of roots and leaves, habitat origin and incubation habitat had almost no effect on changes in [N].

### 4.1 Potential N input in forest and tundra

The potential N input from roots was twofold greater in forest than tundra. Root mass production was threefold greater in forest than in tundra, whereas root [N] was not significantly different between habitats. Our results confirm suggestions that increasing cover of woody functional groups in herbaceous vegetation might stimulate N cycling by increasing potential N input following decomposition of aboveground plant tissue (Buckeridge et al., 2010; Chu & Grogan, 2010), but here we show that greater root mass also contributes to greater inputs in forest than tundra. Increased N cycling may be part of a positive feedback cycle, where enhanced soil N drives increased cover of woody functional groups, increasing soil temperature, stimulating microbial activity, and thus further stimulating nutrient cycling (Buckeridge et al., 2010; Myers‐Smith et al., 2011). Increased rates of N cycling have been also linked to faster soil C turnover (Chapin et al., 2005). On the other hand, woody functional groups might act as C sinks, storing C in their woody stems which have long turnover times and thus negatively feedback on climate warming (Buckeridge et al., 2010; Sturm et al., 2005). The same might be true for roots because roots of woody species often have longer turnover times compared to herbaceous species (Eckstein, Karlsson, & Weih, 1999), and roots in general are an important source of soil C (Kumar, Pandey, & Apurv, 2006).

Root C:N ratio might influence the soil microbial community (De Deyn, Cornelissen, & Bardgett, 2008) and consequently N cycling. Orwin et al. (2010) found that root C:N ratio was negatively correlated with the bacteria: fungi ratio in soil. In our case, the greater root C:N ratio in forest than tundra would indicate a smaller bacteria: fungi ratio, which is often found in soils under woody vegetation (van der Wal, Geydan, Kuyper, & de Boer, 2013; Veen, Sundqvist, Metcalfe, & Wilson, 2015; Veen, Sundqvist, & Wardle, 2015). Although a fungus‐dominated decomposer community can indicate slow nutrient cycling (Wardle et al., 2004), the magnitude of differences between forest and tundra was much greater for potential N input (Figure 1a) than for root C:N (Figure 2g,h), suggesting that root mass and potential N input differences will overwhelm root C:N differences, resulting in faster N cycling beneath forest.

In the present study, the potential input of N from roots varied with diameter and color. Potential N input was greatest in roots with a diameter of 0.2–0.5 mm in both forest and tundra (Figure 2a). This resulted from a greater root mass production of roots with 0.2–0.5 mm diameter (Figure 2c), even though [N] decreased with increasing diameter (Figure 2e). Similar to our results, the potential N input of roots in the northern Great Plains tended to be greatest for roots 0.2–0.5 mm in diameter, caused by greater mass production of these diameter classes (Steinaker & Wilson, 2005). As in our results, [N] decreased with increasing root diameter (Goebel et al., 2011; Sun et al., 2013), in both forest and grassland (Steinaker & Wilson, 2005). In contrast, a study of roots of four temperate woody and herbaceous species showed decreasing [N] with increasing root diameter most strongly for woody species (Pregitzer et al., 1997).

Most importantly, in our study, the overall lack of a significant interaction involving root diameter and habitat suggests that functional groups in forest and tundra have similar distributions of potential N input from roots of different diameters. The greater N input and the lower C:N ratio (Figure 2g) of roots with a smaller diameter in both forest and tundra, together with a missing interaction between root diameter and habitat, suggest that smaller roots possibly contribute to a greater extent to N cycling, and that this pattern does not vary between the functional groups that dominate these habitats. Consequently, any effect of increasing woody plant cover on N input rates is not likely due to variation in root diameter between functional groups.

Here, the potential N input of roots tended to be greater for brown than white roots (Figure 2b), because root mass production tended to be greater for brown than white roots, but significantly so only in tundra (Figure 2d). [N] was greater in brown than white roots, but significantly so only in forest (Figure 2f). Greater [N] in brown roots stands in contrast to a previous study which reported greater [N] in white than brown roots of peach trees (Baldi et al., 2010). The reason for the difference may lie in the differences between study systems (e.g., one species vs. species mixture). The significant interaction between root color and habitat for all variables tested suggests that an increase in the cover of woody vegetation in tundra might alter N cycling due to differences between brown and white roots in potential N input. The greater potential N input of brown compared to white roots in tundra might be balanced out during woody species expansion, because no difference in brown and white roots occurred in forest. In total, differences between forest and tundra in the potential N input of roots to N cycling did not seem to be due to differences in diameter or color classes of roots between the two habitats, because variation among diameter classes was always similar in forest and tundra, and among color classes tended to be similar in forest and tundra (Figure 2).

Our results for potential N contribution are based on live roots rather than actual root litter. Even though previous studies tried to use root litter for their studies (e.g., Freschet, Cornelissen, van Logtestijn, & Aerts, 2010; Freschet et al., 2012), sampling for actual root litter is challenging and subjective (Gordon & Jackson, 2000), and destructive as whole plants where used to recover root litter in the studies of Freschet et al. (2010, 2012) which is not feasible for our study. Our general conclusion of increased potential N input in forest compared to tundra is likely to reflect results of actual root litter because the magnitude of decomposable root biomass might still be much greater in forest than tundra. Studies analyzing differences in N resorption in roots of woody species compared to herbaceous species are lacking, and early studies found no or very low difference in N content of live and dead roots (e.g., Aerts, 1990). In addition, we used the same approach throughout our study ensuring comparability between forest and tundra. Nevertheless, analyzing the resorption potential of roots in forest and tundra during senescence, also considering different plant functional types (e.g., woody deciduous, forbs, and graminoids), is worthwhile but not feasible in the current study.

As in the case of roots, potential N input from leaves was greater in forest than tundra, due to a greater mass production of aboveground tissue in forest compared to tundra, even though leaf [N] was greater in tundra than forest. Although there is evidence for variation in aboveground tissue quality in forest and tundra (Comas & Eissenstat, 2009; Tjoelker, Craine, Wedin, Reich, & Tilman, 2005; Vivanco & Austin, 2006), greater aboveground N input in forest can be expected because mass production in forest exceeds that in an herbaceous habitat by magnitudes (Mendoza‐Ponce & Galicia, 2010; Steinaker & Wilson, 2005; Sturm et al., 2005).

### 4.2 Changes in tissue N concentration during decomposition in forest and tundra

Changes in [N] after 1 year of decomposition varied significantly among tissue types (roots vs. leaves) and, to a lesser extent, between the habitat where decomposition occurred. The habitat origin of tissue types did not influence change in [N]. Woody and herbaceous functional groups can be expected to differ in decomposition dynamics (Wardle et al., 2004), and consequently in change in [N] following decomposition. As noted above, soil under woody vegetation is dominated by fungal communities (Veen, Sundqvist, Metcalfe, et al., 2015; Veen, Sundqvist, & Wardle, 2015), whereas soil under herbaceous vegetation is dominated by bacterial communities (Eskelinen, Stark, & Männistö, 2009) with faster nutrient cycling (van der Wal et al., 2013; Wardle et al., 2004). In our study system, however, the habitat where decomposition occurred had almost no effect on the decomposition of roots and leaves. Only roots >0.2 mm showed a significant difference in the change in [N] between habitats (Figure 3b): in forest, effectively, [N] neither decreased nor increased, whereas in tundra, [N] increased, suggesting that N was accumulated. The initial and after decomposition [N] and C:N ratio did not vary between forest and tundra, suggesting that differences between habitats in change in [N] of roots >0.2 mm are not caused by tissue quality differences between functional groups. A possibility is that the effect of decomposing roots >0.2 mm is delayed due to the fungal‐driven slower decomposition in forest, compared to bacteria‐driven faster decomposition in tundra. Accordingly, long‐term decomposition effects should be tested. Nevertheless, early stages of root and leaf decomposition in herbaceous vegetation might not be affected by the increasing cover of woody vegetation, as the change in [N] during decomposition of almost all tissue types was similar in forest and tundra.

Nitrogen concentration and C:N ratio of roots after decomposition did not vary between forest and tundra, but did vary with root diameter. Root chemistry (i.e., [N], [C], lignin concentration) is one of the most important factors influencing root decomposition (Prieto, Stokes, & Roumet, 2016; Silver & Miya, 2001). A study analyzing two temperate tree species showed that during decomposition roots <0.5 mm lost more N compared to roots >0.5 mm (Sun et al., 2013). Our results indicate the same trend, that smaller roots of ≤0.2 mm lost N, whereas roots >0.2 mm did not contribute or did even accumulate N (Figure 3a,b). Decreasing [N] (Fornara, Tilman, & Hobbie, 2009) as well as N accumulation in early stages of root decomposition has been reported previously (Goebel et al., 2011; Sun et al., 2013). Decomposer communities are strong competitors for N against plants resulting in a short‐term withdrawal (immobilization) of N rather than release of N (mineralization) (Bardgett, Streeter, Cole, & Hartley, 2002; Fornara et al., 2011). Our decomposition experiment lasted for 1 year and presumably reflected mostly immobilization during early stages of decomposition.

The habitat origin had no effect on changes in tissue N during decomposition. This possible lack of home‐field advantage during decomposition was also reported in a study near our study sites analyzing leaf mass loss (Veen, Sundqvist, & Wardle, 2015), but stands in contrast to other studies analyzing the effect of home‐field advantage (Ayres et al., 2009; Gholz et al., 2000; Vivanco & Austin, 2008). Studies to date focusing on home‐field advantage during decomposition were almost entirely done on aboveground tissue, and whether roots show the same effect is unknown. The possible lack of a home‐field advantage might be because we used a mix of species from each habitat, which might mask any species‐specific advantage (Austin, Vivanco, González‐Arzac, & Pérez, 2014; Veen, Sundqvist, & Wardle, 2015). Further, because the [N] and C:N ratio before and after decomposition of both our tissue types (roots and leaves) did not differ between origin habitats, decomposer communities might be expected to be little influenced by tissue from another habitat (Veen, Freschet, Ordonez, & Wardle, 2015). On the other hand, a lack of home‐field advantage could also result from the ability of decomposers of both habitats to decompose a wide range of litter rather than specialization for particular “home” litter (Keiser et al., 2014). Testing for the ability of the decomposer community is beyond the scope of this study but is worth further analysis. Taken together, the increasing cover of woody species into herbaceous vegetation might not affect the release or accumulation of N in roots and leaves during decomposition, although long‐term decomposition effects should be tested.

In our study, we focused on the habitat dependence of the chemical composition and dynamics of nitrogen concentration in roots (and leaves) during their decomposition. To derive from those parameters, the absolute amount of nitrogen released during decomposition one requires additionally information on tissue mass loss, which we could not present here (see Materials and Methods). However, a brief discussion of the interplay of the parameters described here with tissue mass loss can be found in the Supporting Information (“Total N release dynamics”).

## 5 CONCLUSIONS

The potential N input of roots and leaves was greater in forest than in tundra, primarily due to greater production in forest. Greater potential N input in forest than tundra suggests that plant‐associated belowground and aboveground contributions to the N cycle in tundra are likely to be increased by the increasing cover of woody vegetation. Additionally, potential N input varied with root diameter and color, but this variation tended to be similar in forest and tundra, suggesting that increasing cover of woody vegetation in tundra will not alter potential root contributions to N cycling based on differences in root traits. The lack of a habitat origin effect during decomposition for both roots and leaves suggests that decomposition in tundra might not be altered by the increasing cover of woody vegetation. Overall, roots should be included in considerations of differences between forest and herbaceous vegetation in potential plant contributions to soil N cycling, because they reinforce differences between forest and tundra in their leaf production and litter quality.

## DATA ACCESSIBILITY

Data are deposited in the Dryad repository: doi:10.5061/dryad.qg003.

## CONFLICT OF INTEREST

None declared.

## AUTHOR CONTRIBUTIONS

S.T., A.M., and S.D.W. planned and designed the research. S.T. conducted field and laboratory work. S.T. analyzed the data. S.T. wrote the manuscript with contribution from all other authors.

## Supporting information

' Click here for additional data file.
