# Supplementary material for: Potential contributions of root decomposition to the nitrogen cycle in arctic forest and tundra
Source: Ecol Evol. 2017 Nov 15;7(24):11021–32. doi: 10.1002/ece3.3522 (PMC5743615; doi:10.1002/ece3.3522)
Supplement: Supplementary file 1 — ' [file ECE3-7-11021-s001.docx]

**Supporting Information**

“Potential contributions of root decomposition to the nitrogen cycle in arctic forest and tundra”

S. Träger, A. Milbau, and S. D. Wilson

**Supporting Methods**

### Potential N input: aboveground production, N concentration and N input

We calculated the potential N input of aboveground plant tissue in forest and tundra by measuring leaf production of trees and shrubs, herbaceous understorey vegetation in forest, and leaf production of herbaceous vegetation in tundra, and multiplying leaf production with the respective [N] of aboveground tissue.

We examined aboveground leaf production at the forest habitat by collecting leaves of trees, shrubs and herbaceous understorey in 2013 and 2014. For leaves of trees and shrubs we placed three plastic traps (30 x 30 x 25 cm deep; with holes in the bottom to allow drainage) on the forest floor at each location in a triangular design c. 5 m apart. Traps were set out at the beginning of each growing season and sampled biweekly during spring and summer, and weekly during fall. Tree and shrub leaf samples were pooled per location. Production and potential N input of twigs were only 3% and 2% of the total potential N input in forest, respectively (S. Träger, unpublished data), thus, we consider only leaf tissue for further analyses. The understorey aboveground leaf production in forest was measured by cutting herbaceous vegetation in two 10 x 100 cm plots, pooled per location, at the end of the growing season of each year (beginning of September). Plant material of previous years was sorted out according to colour and structure, and understorey production from the current year was retained. Tree, shrub and understorey leaves were dried for 48 h at 60 °C and weighed, respectively. All litter types [leaves from traps, twigs, understory (which include herbaceous leaves)] were collected and analysed separately. At the tundra site, aboveground production was measured by harvesting herbaceous vegetation of two 10 x 100 cm plots pooled per location in fall of each year (beginning of September), sorting out the current year’s production, drying, and weighing.

Sample preparation and determination of total [N] and [C] of leaves from 2013 and 2014 was done as for roots. We determined potential N input of leaf production by multiplying the annual production of aboveground components (tree and shrub leaves and understorey in forest, and herbaceous leaves in tundra) by their respective [N].

### Decomposition experiment: sampling of leaves

Leaves were collected from two subplots (20 x 10 cm) and mixed per location, within 5 m where roots were sampled, resulting in four leaf samples per habitat per area. We collected herbaceous vegetation in forest and tundra, as well as tree leaves in forest, end of August 2014. The two subplots were pooled per location. Leaves with obvious damage from infection or herbivores were excluded. Leaves were chopped to c. 2 cm² pieces, mixed and air dried. One air-dried leaf subsample per location was oven-dried for initial nutrient analysis. Two air-dried leaf subsamples per location were weighed (c. 1 g) and placed in litterbags. One subsample decomposed in its own location (where it was sampled), and the other decomposed in its respective location in the other habitat.

**Supporting Discussion**

### Total N release dynamics

In the main scope of our paper we studied changes in N concentration in roots and leaves in the biomass retained by the sample during decomposition, e.g. “washed out” from the sample through physical/chemical influences or action of biotic factors. To determine the total absolute amount of N released into the environment, however, one would assume that the N contained in the biomass removed from the sample through physical action (i.e. mass loss) is made available to the environment thus constituting a part of the nutrient release flow. Assuming that the initial dry mass of the sample is $m(0)$ and the retained dry mass after time $t$ of decomposition is $m(t)$, the mass loss fraction would be expressed as $L=L(t)=(m\left( 0 \right)-m\left( t \right))/m(0)$. If then the measured [N] in the sample at the beginning of the experiment and after time $t$ of decomposition are $C_{N}(0)$ and $C_{N}\left( t \right)$, respectively, the total N released into the environment after time $t$ of decomposition can be estimated as the sum of N contained in the mass lost by the sample and the N “washed out” of the retained mass as:

$$M_{N,rel}\left( t \right)=\left( m\left( 0 \right)-m\left( t \right) \right)\cdot C_{N}\left( 0 \right)+m(t)\cdot(C_{N}\left( 0 \right)-C_{N}\left( t \right)).$$

Rearranging the elements and substituting $L$ into the equation we obtain $M_{N,rel}\left( t,L\left( t \right),C_{N}\left( 0 \right),C_{N}\left( t \right) \right)$ as a linear functional of $L\left( t \right)$

$$M_{N,rel}\left( t \right)=m\left( 0 \right)\cdot\left( {L(t)\cdot C}_{N}\left( t \right)+(C_{N}\left( 0 \right)-C_{N}\left( t \right)) \right).$$

This shall be important for us since we could not present the mass loss parameter $L$ and with it the exact amount of N released into the environment. We can, however explore the total N release behaviour for different root diameter classes (≤0.2 mm and >0.2 mm) and different habitats as well as for leaves in a reasonable range of possible $L$ values, e.g. between $0$ and $0.5$ (0% to 50% mass loss). Substituting the initial and final [N] values into the above equation, normalizing the values to an initial mass of $m\left( 0 \right)=1g$ we obtain the dependence of the total released N on $L$ as displayed in Fig. S1. Please note, that the calculation does not inform about the efficiency of decomposition.

Fig. S1, displaying total absolute amounts potentially released, confirms the general patterns of change in [N] reported in our study. This becomes especially apparent for roots with a diameter >0.2 mm (thicker roots) and leaves. Thicker roots in tundra tend to accumulate N up to a potential mass loss of 25%, whereas those in forest tend to release N after about 3% mass loss. For leaves, both in forest and tundra, N is accumulated up to a mass loss of 20%, and only during ongoing decomposition and mass loss released. Consequently, our results of changes in [N] are proven to be based within early stages of decomposition with mass loss below 20%. In a study by Solly *et al. (*2014) in a temperate area, fine roots lost between 12% (in forest) and 24% (in grassland) mass within 12 months of decomposition. Our study area in the Arctic is expected to have lower decomposition rates (Freschet *et al.* 2012) validating the presented relationship between forest and tundra in their change in [N] as shown in Fig. S1, below mass loss of 20%.

Combining the results of about twice as much mass loss for herbaceous grassland fine roots than for woody forest species within 12 months of decomposition (Solly *et al.* 2014) with our results of changes in [N] after 12 months of decomposition, we could imply that: Roots >0.2 mm in tundra might not accumulate but loose N, comparable to forest. In contrast, roots ≤0.2 mm in tundra might loose comparable or more N than roots in forest within one year of decomposition. However, the results of Solly *et al.* (2014) were gained in a temperate area where decomposition is likely faster than in our arctic study system. Consequently, our results of effectively no difference in change in [N] in forest and tundra might be projectable to the absolute amounts of N contribution during early stages of decomposition in both systems.

To summarize, our presented results of changes in [N] are feasible and show the likely N contribution of roots and leaves during early stages of decomposition in forest and tundra. The absolute amounts of N released during decomposition constitutes an interesting quantity which is worthwhile to be addressed in future studies.

Solly, E.F., Schöning, I., Boch, S. *et al.* (2014) Factors controlling decomposition rates of fine root litter in temperate forests and grasslands. *Plant and Soil*, **382**, 203-218.

**Supporting Figures**

**
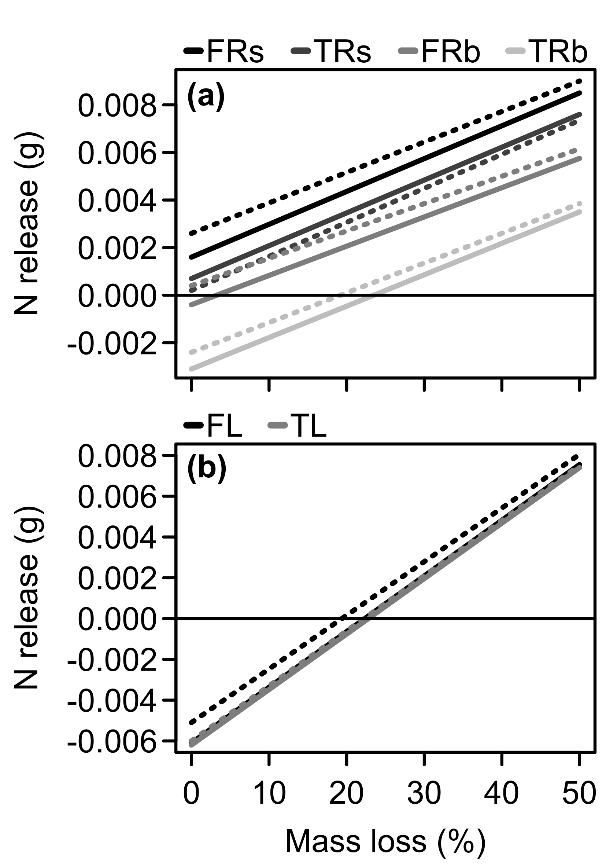
**

**Fig. S1** Relationship between the total amount of N released and tissue mass loss for roots (a) and leaves (b) in forest and tundra when decomposing in an “home” habitat (solid lines) or an “away” habitat (dashed lines). Abbreviations are as follows: F – forest, T – tundra, R – roots, L – leaves, s – roots ≤0.2 mm, b – roots >0.2mm. The relationship is based on the calculation of $M_{N,rel}\left( t \right)=m\left( 0 \right)\cdot\left( {L(t)\cdot C}_{N}\left( t \right)+(C_{N}\left( 0 \right)-C_{N}\left( t \right)) \right)$, with the initial dry mass of the sample, $m(0)$, and the retained dry mass after time $t$ of decomposition, $m(t)$, the mass loss fraction expressed as $L=L(t)=(m\left( 0 \right)-m\left( t \right))/m(0)$, the measured [N] in the sample at the beginning of the experiment, $C_{N}(0)$, and after time $t$ of decomposition $C_{N}\left( t \right)$, and the total amount of N released into the environment after time $t$ of decomposition, $M_{N,rel}\left( t \right)$. The initial mass was normalized to $m\left( 0 \right)=1g$.
